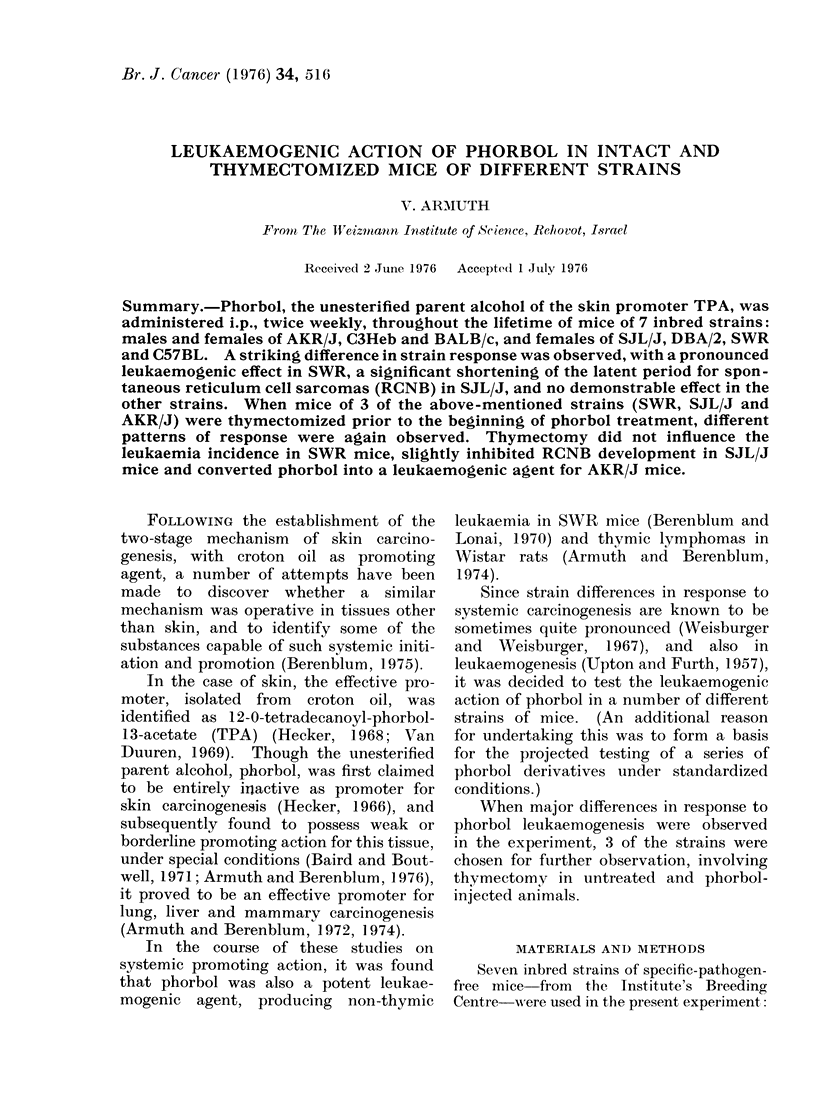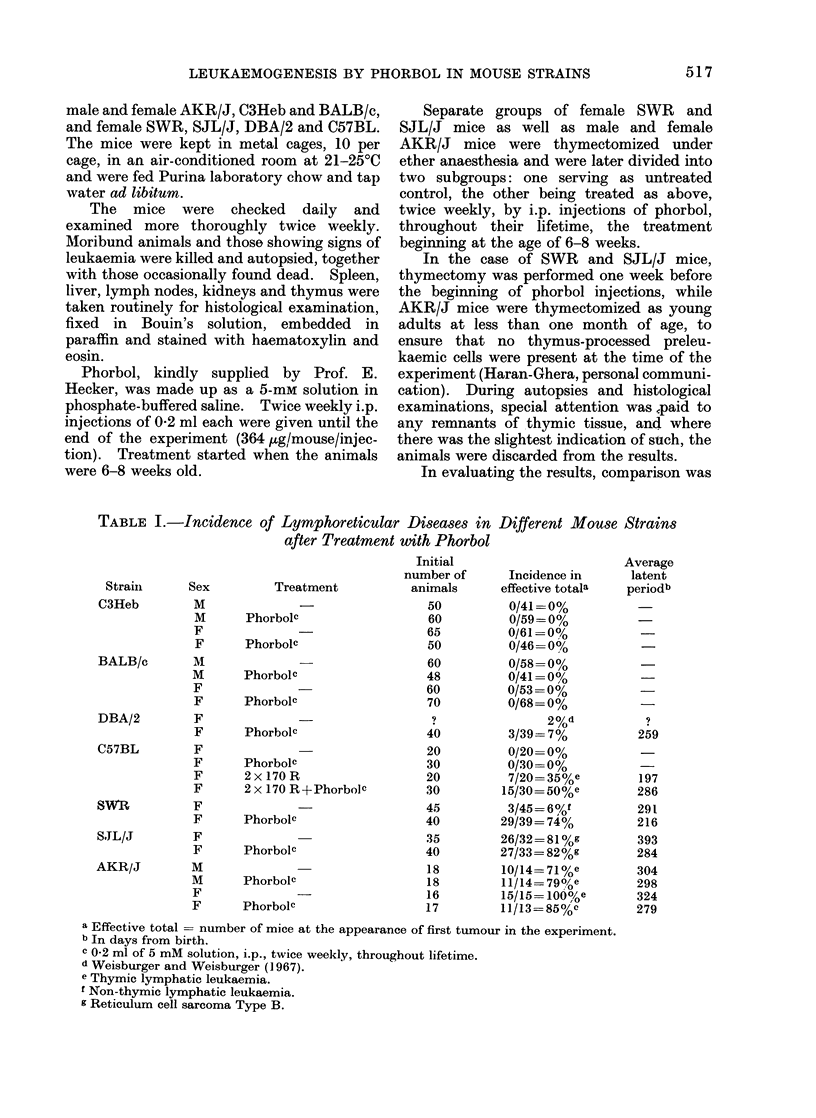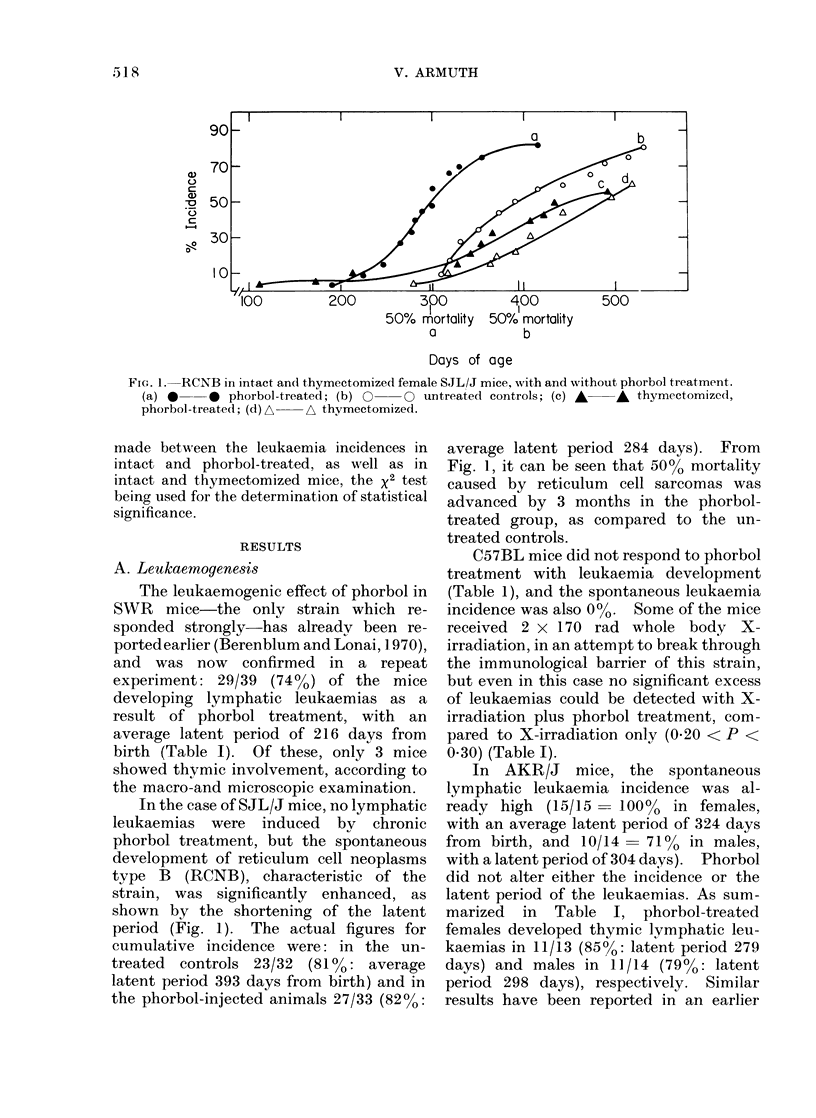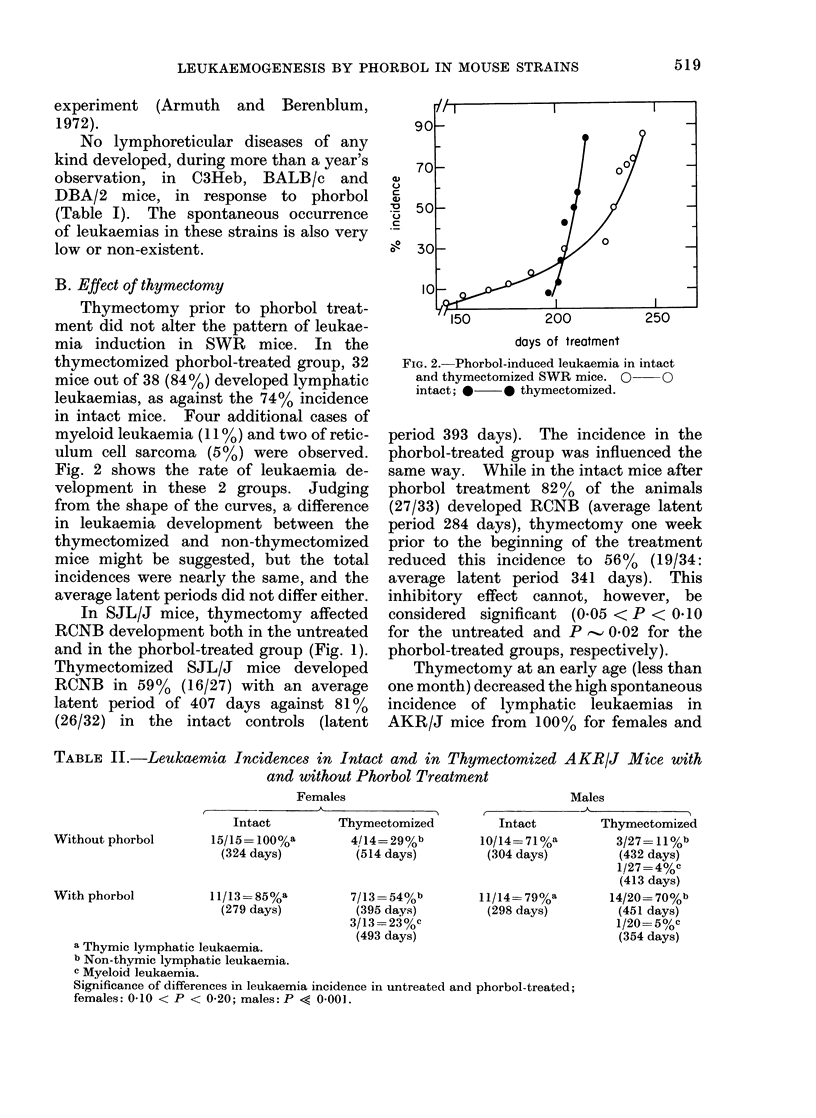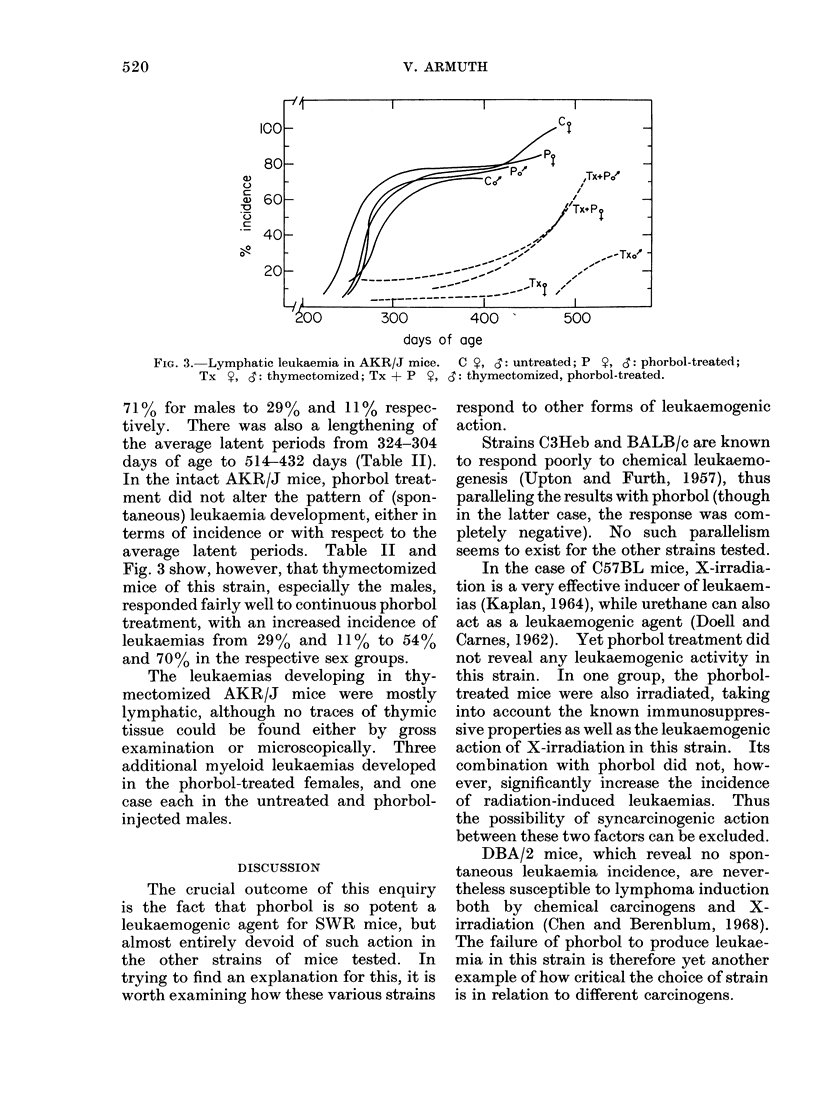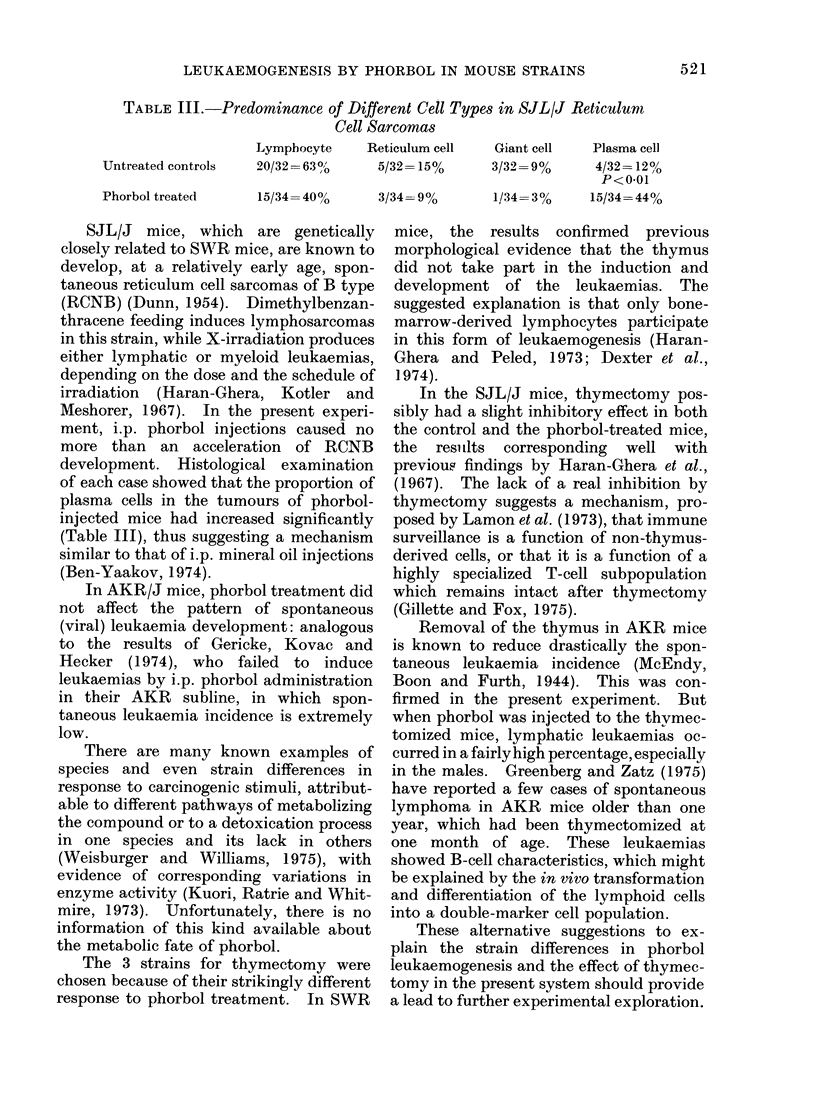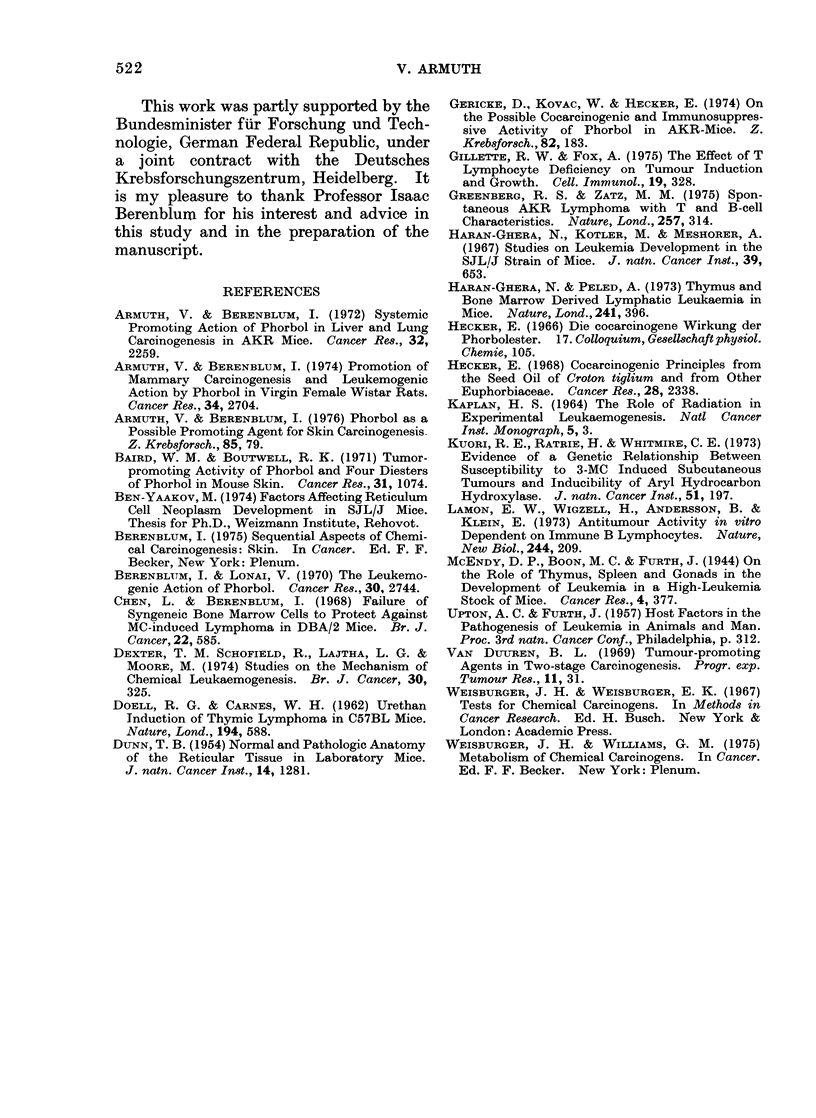# Leukaemogenic action of phorbol in intact and thymectomized mice of different strains.

**DOI:** 10.1038/bjc.1976.206

**Published:** 1976-11

**Authors:** V. Armuth

## Abstract

Phorbol, the unesterified parent alcohol of the skin promoter TPA, was administered i.p., twice weekly, throughout the lifetime of mice of 7 inbred strains: males and females of AKR/J, C3Heb and BALB/c, and females of SJL/J, DBA/2, SWR and C57BL. A striking difference in strain response was observed, with a pronounced leukaemogenic effect in SWR, a signficiant shortening of the latent period for spontaneous reticulum cell sarcomas (RCNB) in SJL/J, and no demonstrable effect in the other strains. When mice of 3 of the above-mentioned strains (SWR, SJL/J and AKR/J) were thymectomized prior to the beginning of phorbol treatment, different patterns of response were again observed. Thymectomy did not influence the leukaemia incidence in SWR mice, slightly inhibited RCNB development in SJL/J mice and converted phorbol into a leukaemogenic agent for AKR/J mice.


*Br. J. Cancer* (1976) **34**, 516

### LEUKAEMOGENIC ACTION OF PHORBOL IN INTACT AND THYMECTOMIZED MICE OF DIFFERENT STRAINS

V. ARMUTH

*From The Weizmann Institute of Science, Rehovot, Israel*

Received 2 June 1976 Accepted 1 July 1976

**Summary.—Phorbol, the unesterified parent alcohol of the skin promoter TPA, was administered i.p., twice weekly, throughout the lifetime of mice of 7 inbred strains: males and females of AKR/J, C3Heb and BALB/c, and females of SJL/J, DBA/2, SWR and C57BL. A striking difference in strain response was observed, with a pronounced leukaemogenic effect in SWR, a significant shortening of the latent period for spontaneous reticulum cell sarcomas (RCNB) in SJL/J, and no demonstrable effect in the other strains. When mice of 3 of the above-mentioned strains (SWR, SJL/J and AKR/J) were thymectomized prior to the beginning of phorbol treatment, different patterns of response were again observed. Thymectomy did not influence the leukaemia incidence in SWR mice, slightly inhibited RCNB development in SJL/J mice and converted phorbol into a leukaemogenic agent for AKR/J mice.**

FOLLOWING the establishment of the two-stage mechanism of skin carcinogenesis, with croton oil as promoting agent, a number of attempts have been made to discover whether a similar mechanism was operative in tissues other than skin, and to identify some of the substances capable of such systemic initiation and promotion (Berenblum, 1975).

In the case of skin, the effective promoter, isolated from croton oil, was identified as 12-0-tetradecanoyl-phorbol-13-acetate (TPA) (Hecker, 1968; Van Duuren, 1969). Though the unesterified parent alcohol, phorbol, was first claimed to be entirely inactive as promoter for skin carcinogenesis (Hecker, 1966), and subsequently found to possess weak or borderline promoting action for this tissue, under special conditions (Baird and Boutwell, 1971; Armuth and Berenblum, 1976), it proved to be an effective promoter for lung, liver and mammary carcinogenesis (Armuth and Berenblum, 1972, 1974).

In the course of these studies on systemic promoting action, it was found that phorbol was also a potent leukaemogenic agent, producing non-thymic leukaemia in SWR mice (Berenblum and Lonai, 1970) and thymic lymphomas in Wistar rats (Armuth and Berenblum, 1974).

Since strain differences in response to systemic carcinogenesis are known to be sometimes quite pronounced (Weisburger and Weisburger, 1967), and also in leukaemogenesis (Upton and Furth, 1957), it was decided to test the leukaemogenic action of phorbol in a number of different strains of mice. (An additional reason for undertaking this was to form a basis for the projected testing of a series of phorbol derivatives under standardized conditions.)

When major differences in response to phorbol leukaemogenesis were observed in the experiment, 3 of the strains were chosen for further observation, involving thymectomy in untreated and phorbol-injected animals.

### MATERIALS AND METHODS

Seven inbred strains of specific-pathogen-free mice—from the Institute's Breeding Centre—were used in the present experiment:

male and female AKR/J, C3Heb and BALB/c, and female SWR, SJL/J, DBA/2 and C57BL. The mice were kept in metal cages, 10 per cage, in an air-conditioned room at 21–25°C and were fed Purina laboratory chow and tap water *ad libitum.*

The mice were checked daily and examined more thoroughly twice weekly. Moribund animals and those showing signs of leukaemia were killed and autopsied, together with those occasionally found dead. Spleen, liver, lymph nodes, kidneys and thymus were taken routinely for histological examination, fixed in Bouin's solution, embedded in paraffin and stained with haematoxylin and eosin.

Phorbol, kindly supplied by Prof. E. Hecker, was made up as a 5-mM solution in phosphate-buffered saline. Twice weekly i.p. injections of 0·2 ml each were given until the end of the experiment (364 $\mu$g/mouse/injection). Treatment started when the animals were 6–8 weeks old.

Separate groups of female SWR and SJL/J mice as well as male and female AKR/J mice were thymectomized under ether anaesthesia and were later divided into two subgroups: one serving as untreated control, the other being treated as above, twice weekly, by i.p. injections of phorbol, throughout their lifetime, the treatment beginning at the age of 6–8 weeks.

In the case of SWR and SJL/J mice, thymectomy was performed one week before the beginning of phorbol injections, while AKR/J mice were thymectomized as young adults at less than one month of age, to ensure that no thymus-processed preleukaemic cells were present at the time of the experiment (Haran-Ghera, personal communication). During autopsies and histological examinations, special attention was paid to any remnants of thymic tissue, and where there was the slightest indication of such, the animals were discarded from the results.

In evaluating the results, comparison was

TABLE I.—*Incidence of Lymphoreticular Diseases in Different Mouse Strains after Treatment with Phorbol*

| Strain | Sex | Treatment | Initial number of animals | Incidence in effective total[a] | Average latent period[b] |
|---|---|---|---|---|---|
| C3Heb | M | — | 50 | 0/41 = 0% | — |
| | M | Phorbol[c] | 60 | 0/59 = 0% | — |
| | F | — | 65 | 0/61 = 0% | — |
| | F | Phorbol[c] | 50 | 0/46 = 0% | — |
| BALB/c | M | — | 60 | 0/58 = 0% | — |
| | M | Phorbol[c] | 48 | 0/41 = 0% | — |
| | F | — | 60 | 0/53 = 0% | — |
| | F | Phorbol[c] | 70 | 0/68 = 0% | — |
| DBA/2 | F | — | ? | 2%[d] | ? |
| | F | Phorbol[c] | 40 | 3/39 = 7% | 259 |
| C57BL | F | — | 20 | 0/20 = 0% | — |
| | F | Phorbol[c] | 30 | 0/30 = 0% | — |
| | F | 2 × 170 R | 20 | 7/20 = 35%[e] | 197 |
| | F | 2 × 170 R + Phorbol[c] | 30 | 15/30 = 50%[e] | 286 |
| SWR | F | — | 45 | 3/45 = 6%[f] | 291 |
| | F | Phorbol[c] | 40 | 29/39 = 74% | 216 |
| SJL/J | F | — | 35 | 26/32 = 81%[g] | 393 |
| | F | Phorbol[c] | 40 | 27/33 = 82%[g] | 284 |
| AKR/J | M | — | 18 | 10/14 = 71%[e] | 304 |
| | M | Phorbol[c] | 18 | 11/14 = 79%[e] | 298 |
| | F | — | 16 | 15/15 = 100%[e] | 324 |
| | F | Phorbol[c] | 17 | 11/13 = 85%[c] | 279 |

[a] Effective total = number of mice at the appearance of first tumour in the experiment.
[b] In days from birth.
[c] 0·2 ml of 5 mM solution, i.p., twice weekly, throughout lifetime.
[d] Weisburger and Weisburger (1967).
[e] Thymic lymphatic leukaemia.
[f] Non-thymic lymphatic leukaemia.
[g] Reticulum cell sarcoma Type B.

FIG. 1.—RCNB in intact and thymectomized female SJL/J mice, with and without phorbol treatment. (a) ●——● phorbol-treated; (b) ○——○ untreated controls; (c) ▲——▲ thymectomized, phorbol-treated; (d) △——△ thymectomized.

made between the leukaemia incidences in intact and phorbol-treated, as well as in intact and thymectomized mice, the $\chi^2$ test being used for the determination of statistical significance.

### RESULTS

### A. *Leukaemogenesis*

The leukaemogenic effect of phorbol in SWR mice—the only strain which responded strongly—has already been reported earlier (Berenblum and Lonai, 1970), and was now confirmed in a repeat experiment: 29/39 (74%) of the mice developing lymphatic leukaemias as a result of phorbol treatment, with an average latent period of 216 days from birth (Table I). Of these, only 3 mice showed thymic involvement, according to the macro-and microscopic examination.

In the case of SJL/J mice, no lymphatic leukaemias were induced by chronic phorbol treatment, but the spontaneous development of reticulum cell neoplasms type B (RCNB), characteristic of the strain, was significantly enhanced, as shown by the shortening of the latent period (Fig. 1). The actual figures for cumulative incidence were: in the untreated controls 23/32 (81%: average latent period 393 days from birth) and in the phorbol-injected animals 27/33 (82%: average latent period 284 days). From Fig. 1, it can be seen that 50% mortality caused by reticulum cell sarcomas was advanced by 3 months in the phorbol-treated group, as compared to the untreated controls.

C57BL mice did not respond to phorbol treatment with leukaemia development (Table 1), and the spontaneous leukaemia incidence was also 0%. Some of the mice received 2 × 170 rad whole body X-irradiation, in an attempt to break through the immunological barrier of this strain, but even in this case no significant excess of leukaemias could be detected with X-irradiation plus phorbol treatment, compared to X-irradiation only ($0{\cdot}20 < P < 0{\cdot}30$) (Table I).

In AKR/J mice, the spontaneous lymphatic leukaemia incidence was already high (15/15 = 100% in females, with an average latent period of 324 days from birth, and 10/14 = 71% in males, with a latent period of 304 days). Phorbol did not alter either the incidence or the latent period of the leukaemias. As summarized in Table I, phorbol-treated females developed thymic lymphatic leukaemias in 11/13 (85%: latent period 279 days) and males in 11/14 (79%: latent period 298 days), respectively. Similar results have been reported in an earlier experiment (Armuth and Berenblum, 1972).

No lymphoreticular diseases of any kind developed, during more than a year's observation, in C3Heb, BALB/c and DBA/2 mice, in response to phorbol (Table I). The spontaneous occurrence of leukaemias in these strains is also very low or non-existent.

B. *Effect of thymectomy*

Thymectomy prior to phorbol treatment did not alter the pattern of leukaemia induction in SWR mice. In the thymectomized phorbol-treated group, 32 mice out of 38 (84%) developed lymphatic leukaemias, as against the 74% incidence in intact mice. Four additional cases of myeloid leukaemia (11%) and two of reticulum cell sarcoma (5%) were observed. Fig. 2 shows the rate of leukaemia development in these 2 groups. Judging from the shape of the curves, a difference in leukaemia development between the thymectomized and non-thymectomized mice might be suggested, but the total incidences were nearly the same, and the average latent periods did not differ either.

In SJL/J mice, thymectomy affected RCNB development both in the untreated and in the phorbol-treated group (Fig. 1). Thymectomized SJL/J mice developed RCNB in 59% (16/27) with an average latent period of 407 days against 81% (26/32) in the intact controls (latent

Fig. 2.—Phorbol-induced leukaemia in intact and thymectomized SWR mice. ○——○ intact; ●——● thymectomized.

period 393 days). The incidence in the phorbol-treated group was influenced the same way. While in the intact mice after phorbol treatment 82% of the animals (27/33) developed RCNB (average latent period 284 days), thymectomy one week prior to the beginning of the treatment reduced this incidence to 56% (19/34: average latent period 341 days). This inhibitory effect cannot, however, be considered significant ($0{\cdot}05 < P < 0{\cdot}10$ for the untreated and $P \sim 0{\cdot}02$ for the phorbol-treated groups, respectively).

Thymectomy at an early age (less than one month) decreased the high spontaneous incidence of lymphatic leukaemias in AKR/J mice from 100% for females and

TABLE II.—*Leukaemia Incidences in Intact and in Thymectomized AKR/J Mice with and without Phorbol Treatment*

| | Females | | Males | |
|---|---|---|---|---|
| | Intact | Thymectomized | Intact | Thymectomized |
| Without phorbol | 15/15=100%[a] (324 days) | 4/14=29%[b] (514 days) | 10/14=71%[a] (304 days) | 3/27=11%[b] (432 days) 1/27=4%[c] (413 days) |
| With phorbol | 11/13=85%[a] (279 days) | 7/13=54%[b] (395 days) 3/13=23%[c] (493 days) | 11/14=79%[a] (298 days) | 14/20=70%[b] (451 days) 1/20=5%[c] (354 days) |

[a] Thymic lymphatic leukaemia.
[b] Non-thymic lymphatic leukaemia.
[c] Myeloid leukaemia.
Significance of differences in leukaemia incidence in untreated and phorbol-treated; females: $0{\cdot}10 < P < 0{\cdot}20$; males: $P \ll 0{\cdot}001$.

FIG. 3.—Lymphatic leukaemia in AKR/J mice. C ♀, ♂: untreated; P ♀, ♂: phorbol-treated; Tx ♀, ♂: thymectomized; Tx + P ♀, ♂: thymectomized, phorbol-treated.

71% for males to 29% and 11% respectively. There was also a lengthening of the average latent periods from 324–304 days of age to 514–432 days (Table II). In the intact AKR/J mice, phorbol treatment did not alter the pattern of (spontaneous) leukaemia development, either in terms of incidence or with respect to the average latent periods. Table II and Fig. 3 show, however, that thymectomized mice of this strain, especially the males, responded fairly well to continuous phorbol treatment, with an increased incidence of leukaemias from 29% and 11% to 54% and 70% in the respective sex groups.

The leukaemias developing in thymectomized AKR/J mice were mostly lymphatic, although no traces of thymic tissue could be found either by gross examination or microscopically. Three additional myeloid leukaemias developed in the phorbol-treated females, and one case each in the untreated and phorbol-injected males.

### DISCUSSION

The crucial outcome of this enquiry is the fact that phorbol is so potent a leukaemogenic agent for SWR mice, but almost entirely devoid of such action in the other strains of mice tested. In trying to find an explanation for this, it is worth examining how these various strains respond to other forms of leukaemogenic action.

Strains C3Heb and BALB/c are known to respond poorly to chemical leukaemogenesis (Upton and Furth, 1957), thus paralleling the results with phorbol (though in the latter case, the response was completely negative). No such parallelism seems to exist for the other strains tested.

In the case of C57BL mice, X-irradiation is a very effective inducer of leukaemias (Kaplan, 1964), while urethane can also act as a leukaemogenic agent (Doell and Carnes, 1962). Yet phorbol treatment did not reveal any leukaemogenic activity in this strain. In one group, the phorbol-treated mice were also irradiated, taking into account the known immunosuppressive properties as well as the leukaemogenic action of X-irradiation in this strain. Its combination with phorbol did not, however, significantly increase the incidence of radiation-induced leukaemias. Thus the possibility of syncarcinogenic action between these two factors can be excluded.

DBA/2 mice, which reveal no spontaneous leukaemia incidence, are nevertheless susceptible to lymphoma induction both by chemical carcinogens and X-irradiation (Chen and Berenblum, 1968). The failure of phorbol to produce leukaemia in this strain is therefore yet another example of how critical the choice of strain is in relation to different carcinogens.

TABLE III.—*Predominance of Different Cell Types in SJL/J Reticulum Cell Sarcomas*

| | Lymphocyte | Reticulum cell | Giant cell | Plasma cell |
|---|---|---|---|---|
| Untreated controls | 20/32=63% | 5/32=15% | 3/32=9% | 4/32=12% |
| | | | | $P<0{\cdot}01$ |
| Phorbol treated | 15/34=40% | 3/34=9% | 1/34=3% | 15/34=44% |

SJL/J mice, which are genetically closely related to SWR mice, are known to develop, at a relatively early age, spontaneous reticulum cell sarcomas of B type (RCNB) (Dunn, 1954). Dimethylbenzanthracene feeding induces lymphosarcomas in this strain, while X-irradiation produces either lymphatic or myeloid leukaemias, depending on the dose and the schedule of irradiation (Haran-Ghera, Kotler and Meshorer, 1967). In the present experiment, i.p. phorbol injections caused no more than an acceleration of RCNB development. Histological examination of each case showed that the proportion of plasma cells in the tumours of phorbol-injected mice had increased significantly (Table III), thus suggesting a mechanism similar to that of i.p. mineral oil injections (Ben-Yaakov, 1974).

In AKR/J mice, phorbol treatment did not affect the pattern of spontaneous (viral) leukaemia development: analogous to the results of Gericke, Kovac and Hecker (1974), who failed to induce leukaemias by i.p. phorbol administration in their AKR subline, in which spontaneous leukaemia incidence is extremely low.

There are many known examples of species and even strain differences in response to carcinogenic stimuli, attributable to different pathways of metabolizing the compound or to a detoxication process in one species and its lack in others (Weisburger and Williams, 1975), with evidence of corresponding variations in enzyme activity (Kuori, Ratrie and Whitmire, 1973). Unfortunately, there is no information of this kind available about the metabolic fate of phorbol.

The 3 strains for thymectomy were chosen because of their strikingly different response to phorbol treatment. In SWR mice, the results confirmed previous morphological evidence that the thymus did not take part in the induction and development of the leukaemias. The suggested explanation is that only bone-marrow-derived lymphocytes participate in this form of leukaemogenesis (Haran-Ghera and Peled, 1973; Dexter *et al.*, 1974).

In the SJL/J mice, thymectomy possibly had a slight inhibitory effect in both the control and the phorbol-treated mice, the results corresponding well with previous findings by Haran-Ghera *et al.*, (1967). The lack of a real inhibition by thymectomy suggests a mechanism, proposed by Lamon *et al.* (1973), that immune surveillance is a function of non-thymus-derived cells, or that it is a function of a highly specialized T-cell subpopulation which remains intact after thymectomy (Gillette and Fox, 1975).

Removal of the thymus in AKR mice is known to reduce drastically the spontaneous leukaemia incidence (McEndy, Boon and Furth, 1944). This was confirmed in the present experiment. But when phorbol was injected to the thymectomized mice, lymphatic leukaemias occurred in a fairly high percentage, especially in the males. Greenberg and Zatz (1975) have reported a few cases of spontaneous lymphoma in AKR mice older than one year, which had been thymectomized at one month of age. These leukaemias showed B-cell characteristics, which might be explained by the *in vivo* transformation and differentiation of the lymphoid cells into a double-marker cell population.

These alternative suggestions to explain the strain differences in phorbol leukaemogenesis and the effect of thymectomy in the present system should provide a lead to further experimental exploration.

This work was partly supported by the Bundesminister für Forschung und Technologie, German Federal Republic, under a joint contract with the Deutsches Krebsforschungszentrum, Heidelberg. It is my pleasure to thank Professor Isaac Berenblum for his interest and advice in this study and in the preparation of the manuscript.

### REFERENCES

Armuth, V. & Berenblum, I. (1972) Systemic Promoting Action of Phorbol in Liver and Lung Carcinogenesis in AKR Mice. *Cancer Res.*, **32**, 2259.

Armuth, V. & Berenblum, I. (1974) Promotion of Mammary Carcinogenesis and Leukemogenic Action by Phorbol in Virgin Female Wistar Rats. *Cancer Res.*, **34**, 2704.

Armuth, V. & Berenblum, I. (1976) Phorbol as a Possible Promoting Agent for Skin Carcinogenesis. *Z. Krebsforsch.*, **85**, 79.

Baird, W. M. & Boutwell, R. K. (1971) Tumor-promoting Activity of Phorbol and Four Diesters of Phorbol in Mouse Skin. *Cancer Res.*, **31**, 1074.

Ben-Yaakov, M. (1974) Factors Affecting Reticulum Cell Neoplasm Development in SJL/J Mice. Thesis for Ph.D., Weizmann Institute, Rehovot.

Berenblum, I. (1975) Sequential Aspects of Chemical Carcinogenesis: Skin. In *Cancer*. Ed. F. F. Becker, New York: Plenum.

Berenblum, I. & Lonai, V. (1970) The Leukemogenic Action of Phorbol. *Cancer Res.*, **30**, 2744.

Chen, L. & Berenblum, I. (1968) Failure of Syngeneic Bone Marrow Cells to Protect Against MC-induced Lymphoma in DBA/2 Mice. *Br. J. Cancer*, **22**, 585.

Dexter, T. M. Schofield, R., Lajtha, L. G. & Moore, M. (1974) Studies on the Mechanism of Chemical Leukaemogenesis. *Br. J. Cancer*, **30**, 325.

Doell, R. G. & Carnes, W. H. (1962) Urethan Induction of Thymic Lymphoma in C57BL Mice. *Nature, Lond.*, **194**, 588.

Dunn, T. B. (1954) Normal and Pathologic Anatomy of the Reticular Tissue in Laboratory Mice. *J. natn. Cancer Inst.*, **14**, 1281.

Gericke, D., Kovac, W. & Hecker, E. (1974) On the Possible Cocarcinogenic and Immunosuppressive Activity of Phorbol in AKR-Mice. *Z. Krebsforsch.*, **82**, 183.

Gillette, R. W. & Fox, A. (1975) The Effect of T Lymphocyte Deficiency on Tumour Induction and Growth. *Cell. Immunol.*, **19**, 328.

Greenberg, R. S. & Zatz, M. M. (1975) Spontaneous AKR Lymphoma with T and B-cell Characteristics. *Nature, Lond.*, **257**, 314.

Haran-Ghera, N., Kotler, M. & Meshorer, A. (1967) Studies on Leukemia Development in the SJL/J Strain of Mice. *J. natn. Cancer Inst.*, **39**, 653.

Haran-Ghera, N. & Peled, A. (1973) Thymus and Bone Marrow Derived Lymphatic Leukaemia in Mice. *Nature, Lond.*, **241**, 396.

Hecker, E. (1966) Die cocarcinogene Wirkung der Phorbolester. 17. *Colloquium, Gesellschaft physiol. Chemie*, 105.

Hecker, E. (1968) Cocarcinogenic Principles from the Seed Oil of *Croton tiglium* and from Other Euphorbiaceae. *Cancer Res.*, **28**, 2338.

Kaplan, H. S. (1964) The Role of Radiation in Experimental Leukaemogenesis. *Natl Cancer Inst. Monograph*, **5**, 3.

Kuori, R. E., Ratrie, H. & Whitmire, C. E. (1973) Evidence of a Genetic Relationship Between Susceptibility to 3-MC Induced Subcutaneous Tumours and Inducibility of Aryl Hydrocarbon Hydroxylase. *J. natn. Cancer Inst.*, **51**, 197.

Lamon, E. W., Wigzell, H., Andersson, B. & Klein, E. (1973) Antitumour Activity *in vitro* Dependent on Immune B Lymphocytes. *Nature, New Biol.*, **244**, 209.

McEndy, D. P., Boon, M. C. & Furth, J. (1944) On the Role of Thymus, Spleen and Gonads in the Development of Leukemia in a High-Leukemia Stock of Mice. *Cancer Res.*, **4**, 377.

Upton, A. C. & Furth, J. (1957) Host Factors in the Pathogenesis of Leukemia in Animals and Man. *Proc. 3rd natn. Cancer Conf.*, Philadelphia, p. 312.

Van Duuren, B. L. (1969) Tumour-promoting Agents in Two-stage Carcinogenesis. *Progr. exp. Tumour Res.*, **11**, 31.

Weisburger, J. H. & Weisburger, E. K. (1967) Tests for Chemical Carcinogens. In *Methods in Cancer Research*. Ed. H. Busch. New York & London: Academic Press.

Weisburger, J. H. & Williams, G. M. (1975) Metabolism of Chemical Carcinogens. In *Cancer*. Ed. F. F. Becker. New York: Plenum.